# Concurrent Conditions in Patients with Chronic Constipation: A Population–Based Study

**DOI:** 10.1371/journal.pone.0042910

**Published:** 2012-10-12

**Authors:** Gaurav Arora, Ajitha Mannalithara, Alka Mithal, George Triadafilopoulos, Gurkirpal Singh

**Affiliations:** 1 Division of Digestive and Liver Diseases, University of Texas Southwestern Medical Center, Dallas, Texas, United States of America; 2 Division of Gastroenterology and Hepatology, Stanford University School of Medicine, Stanford, California, United States of America; 3 Division of Epidemiology, Institute of Clinical Outcomes Research and Education, Palo Alto, California, United States of America; Ulm University, Germany

## Abstract

**Background:**

Chronic constipation (CC) is a common condition but its concurrent conditions are not well characterized. We measured the prevalence and risk of developing 15 pre–specified concurrent conditions in patients with CC.

**Methods:**

Retrospective cohort study using the Medicaid database of California, utilizing ICD-9 codes for detection of cases (CC), controls (patients with GERD) and concurrent conditions. Study period was 01/01/1995 to 06/30/2005. Index date was the date 3 months before the first physician visit for CC. Pre-index time (12 months) was compared to post-index time (12 months) to assess the association of every concurrent condition within each cohort. To account for ascertainment bias, an adjusted odds ratio was calculated by comparing the odds ratio for every concurrent condition in the CC cohort to that in the GERD cohort.

**Results:**

147,595 patients with CC (mean age 54.2 years; 69.7% women; 36.2% white) and 142,086 patients with GERD (mean age 56.3 years; 65.3% women; 41.6% white) were evaluated. The most prevalent concurrent conditions with CC were hemorrhoids (7.6%), diverticular disease (5.9%), ano–rectal hemorrhage (4.7%), irritable bowel syndrome (3.5%) and fecal impaction (2%). When adjusted for ascertainment bias, the most notable associations with CC were Hirschsprung's disease, fecal impaction and ano-rectal conditions such as fissure, fistula, hemorrhage and ulcers.

**Conclusion:**

Chronic constipation is associated with several concurrent conditions of variable risk and prevalence. To reduce the overall burden of CC, these concurrent conditions need to be addressed.

## Introduction

Constipation is one of the most common digestive problems in North America, with an estimated prevalence between 2% and 27%. [1] For many patients, constipation-associated symptoms are chronic and last for several weeks to several years. [2] The cost of evaluating and treating constipation is significant. Each year in the United States alone, approximately 2.5 million people consult a physician for constipation [3], and approximately 92,000 are hospitalized. [4] Based on an analysis of 3 national surveys in 2001, the annual costs associated with medical care for constipation total $235 million. [5] In a previous study, we evaluated total costs of care of constipation in the California Medicaid program (Medi–Cal). [6] Patients with constipation often experience co-morbid conditions. In our previous study, the most common co-morbidity (amongst the 105,130 patients who saw a physician at least once for constipation) was hemorrhoids, which occurred in 5,657 (5.4%) patients within 1 year of the first visit for constipation. This was followed in frequency by irritable bowel syndrome (3,597 [3.42%] patients) and fecal impaction or intestinal obstruction (2,288 [2.2%] patients). These additional illnesses can further increase the cost of caring for this patient population. We could not establish a relationship between constipation and any of these concurrent conditions, as our study was not designed for that purpose; it is possible that the association between constipation and its concurrent conditions could simply reflect a “detection” or “ascertainment” bias. A concurrent condition could have been identified simply because a patient was examined by a physician, even if that condition was not related to the reason for physician visit. With that in mind, we designed the present study with a control group and compared the “before-after” risk for every concurrent condition in the constipation group, to that in the control group.

## Methods

### Data Source

We used the research database of Medi-Cal, the Medicaid program for the state of California. [7] Medi–Cal provides health care coverage for low-income and disabled individuals who lack health insurance; it covers more than 7 million persons and is the largest state Medicaid program in the United States. Its claims and administrative database are a key source of information for a variety of research efforts related to health care costs, resource utilization, quality, and effectiveness. The Medi–Cal Research Database has information on an estimated 100 million patient-years from 1995–2005 and allows linkage of records to medical and pharmacy claims. Medical claims or encounter records flow into the database from all health care sites (inpatient hospital, outpatient hospital, emergency room, physician's office, outpatient surgery center, *etc*.) for virtually all types of services provided to enrolees, including specialty, preventive and office-based treatments. Each facility service record contains information on diagnoses, recorded with the International Classification of Diseases, Ninth Revision (ICD-9) diagnosis codes, and procedures recorded with ICD-9 procedure codes, Current Procedural Terminology (CPT) or Health Care Financing Agency (HCFA) Common Procedure Coding System (HCPCS) codes. The data in these databases undergo regular audits to ensure quality control. A recently published audit of Medi-Cal claims found that 96.4% were medically necessary, billed appropriately, and were in concordance with the data in the claims files. [8]

### Study Design, Period and Outcomes

A retrospective cohort design was used. This study covered the time period from January 1^st^, 1995 to June 30^th^, 2005. Each person's observation period began on the day he or she joined Medi–Cal or January 1^st^, 1995, whichever occurred later. Observation continued until the earliest of the termination of Medi–Cal eligibility or June 30^th^, 2005. The primary objective of this study was to measure the prevalence of and ascertain the risk of developing 15 pre-specified concurrent conditions (*Table 1*) in patients with chronic constipation. The conditions were chosen based on a review of the existing literature and also on the basis of experience of one of the investigators (G.T.) in treating patients with CC. We did not factor in our analysis any treatments that might have been given for the treatment of CC.

**Table 1:** List of primary study outcomes.

Concurrent Condition of Constipation	ICD-9 Codes
Anal fissure	565.0
Anal fistula	565.1
Ano–rectal hemorrhage	569.3
Ano–rectal ulcers	569.41
Crohn's disease	555
Diverticular Disease	562
Fecal impaction	560.39
Fecal incontinence	787.6
Hemorrhoids	455.0–455.9
Hirschsprung's Disease	751.3
Irritable bowel syndrome	564.1
Malignant neoplasm of colon	153.0–153.9, V1006
Rectal prolapse	569.1
Ulcerative colitis	556.0–556.6, 556.8,556.9
Volvulus	560.2

### Cohort Definition

The study cohort included subjects ≥18 years of age with a diagnosis of chronic constipation (defined by an ICD–9 code of 564.0×). Index date was defined as the date 3 months before the first physician visit for chronic constipation. The subjects were required to have at least 12 months enrollment in Medi–Cal prior to the index date and at least 12 months subsequent. The “pre–index” time was 12 months before and “post–index” time was 12 months after the index date. It was assumed that a patient may have had constipation for a period of 3 months before seeking medical attention.

The control cohort was comprised of individuals diagnosed with an unrelated illness–gastro-esophageal reflux disease (GERD). Using the Montreal definition for GERD, constipation is not an associated condition (like laryngitis, chronic cough, etc). [9] There may be some overlap but it is not pathophysiologically related. We chose GERD because of its chronicity, similar to constipation. They had to be ≥18 years of age with a diagnosis of GERD (ICD-9 codes 787.1, 530.1, 530.2 or 530.3) made at the first physician visit. The enrollment criteria were the same as the study cohort. The “pre–index” and “post–index” time periods were also defined in the same fashion as for the study cohort. Similarly, it was assumed that a patient could have had GERD for a period of 3 months before seeking medical attention.

### Rationale for selection of GERD as the reference group

The necessity of a control group has already been discussed. A control group is needed to document whether an increased odds ratio of “post–index” *vs*. “pre–index” time periods for a particular concurrent condition is simply a result of an increased chance of detection because of a physician visit. An appropriate control group would have similar clinical care patterns as the study group so that this detection bias can be controlled. There are several similarities in the medical management of GERD and constipation. Patients with both conditions are often symptomatic for several months before seeking medical attention. Both conditions are related to the gastrointestinal tract, but are often managed by primary care physicians before being referred to a gastroenterologist.

### Statistical Analysis

The association of every concurrent condition with constipation (or GERD) was assessed by calculating an odds ratio from the probability of occurrence of the condition after the diagnosis of constipation (or GERD) compared to the probability of occurrence of the concurrent condition in the study period before the diagnosis of constipation (or GERD). Three separate pre-specified statistical analyses were performed for each condition: comparison of “post–index” proportion to “pre–index” proportion as an odds ratio in the GERD cohort; comparison of “post–index” proportion to “pre–index” proportion as an odds ratio in the constipation cohort; and comparison of the odds ratios of “post–index vs. pre–index constipation” to “post–index vs. pre–index GERD” as another odds ratio, termed “adjusted–odds ratio”; this last comparison was done to control for the effect of detection bias. To compare the odds ratios in order to derive the “adjusted–odds ratio” and calculate its confidence interval, we used a method recently described by Friedrich *et al*. [10] For all odds ratios, 95% confidence intervals are reported. All analyses were done using SAS 9.1 (Cary, North Carolina, USA).

## Results

A total of 147,595 patients with constipation (mean age 54.2 years; 69.7% women; 36.2% white) and 142,086 patients with GERD (mean age 56.3 years; 65.3% women; 41.6% white) formed the study and control cohorts, respectively (*Table 2*). Results are presented as “pre–index” and “post–index” (counts and percentages) as well as odds ratios (comparing “pre–index” and “post–index” time–periods) for the association of every concurrent condition with GERD and constipation (*Table 3*). The adjusted–ratio is the association of every concurrent condition with constipation when adjusted for the association with GERD. Overall, there were significant differences in the odds ratios of most concurrent conditions between the GERD and constipation groups.

**Table 2:** Demographic characteristics of the control and study cohorts.

	GERD (control)	Constipation (study)
**No. of subjects**	142,086	147,595
**Age (mean [SD])**	56.3 (17.6)	54.2 (20.2)
**Gender (n [%])**		
Women	92844 (65.3)	102940 (69.7)
Men	49242 (34.7)	44655 (30.3)
**Race/Ethnicity (n [%])**		
Caucasian	59147 (41.6)	53483 (36.2)
Black	14171 (10.0)	15826 (10.7)
Hispanic	28057 (19.7)	36835 (25.0)
Asian	20474 (14.4)	21544 (14.6)
Other	5408 (3.8)	5790 (3.9)
Missing	14829 (10.4)	14117 (9.6)

**Table 3:** Association of concurrent conditions with GERD and chronic constipation and the adjusted–ratio.

	GERD (N = 142,086)			Constipation (N = 147,595)			
Concurrent condition	Pre–index n (%)	Post–index n (%)	Odds Ratio (95% CI)	Pre–index n (%)	Post–index n (%)	Odds Ratio (95% CI)	Adjusted-ratio* (95% CI)
**Anal Fissure**	277 (0.19)	344 (0.24)	1.24 (1.06–1.46)	303 (0.21)	921 (0.62)	3.05 (2.68–3.48)	2.46 (2.12–2.84)
**Anal Fistula**	106 (0.07)	139 (0.10)	1.31 (1.02–1.69)	96 (0.07)	216 (0.15)	2.25 (1.77–2.86)	1.72 (1.37–2.15)
**Ano–rectal hemorrhage**	2589 (1.82)	5385 (3.79)	2.12 (2.02–2.23)	2469 (1.67)	6928 (4.69)	2.89 (2.76–3.03)	1.36 (1.30–1.43)
**Ano–rectal ulcers**	77 (0.05)	151 (0.11)	1.96 (1.49–2.58)	50 (0.03)	207 (0.14)	4.14 (3.04–5.64)	2.11 (1.66–2.69)
**Crohn's disease**	412 (0.29)	714 (0.50)	1.74 (1.54–1.96)	275 (0.19)	456 (0.31)	1.66 (1.43–1.93)	0.96 (0.85–1.07)
**Diverticular Disease**	3778 (2.66)	9732 (6.85)	2.69 (2.59–2.80)	3241 (2.20)	8752 (5.93)	2.81 (2.69–2.92)	1.04 (1.00–1.08)
**Fecal Impaction**	351 (0.25)	611 (0.43)	1.74 (1.53–1.99)	550 (0.37)	3022 (2.05)	5.59 (5.10–6.12)	3.20 (2.83–3.62)
**Fecal incontinence**	226 (0.16)	334 (0.24)	1.48 (1.25–1.75)	369 (0.25)	632 (0.43)	1.72 (1.51–1.95)	1.16 (0.99–1.35)
**Hemorrhoids**	3942 (2.77)	8770 (6.17)	2.31 (2.22–2.40)	4139 (2.80)	11267 (7.63)	2.86 (2.76–2.97)	1.24 (1.20–1.30)
**Hirschsprung's disease**	10 (0.01)	10 (0.01)	1.00 (0.42–2.40)	12 (0.01)	53 (0.04)	4.42 (2.36–8.27)	4.42 (2.46–7.92)
**Irritable bowel syndrome**	2653 (1.87)	5093 (3.58)	1.95 (1.86–2.05)	2394 (1.62)	5162 (3.50)	2.20 (2.09–2.31)	1.12 (1.07–1.18)
**Malignant neoplasm of colon**	504 (0.35)	955 (0.67)	1.90 (1.71–2.12)	582 (0.39)	1282 (0.87)	2.21 (2.01–2.44)	1.16 (1.05–1.30)
**Rectal prolapse**	78 (0.05)	111 (0.08)	1.42 (1.07–1.90)	124 (0.08)	288 (0.20)	2.33 (1.88–2.87)	1.63 (1.27–2.10)
**Ulcerative colitis**	419 (0.29)	962 (0.68)	2.30 (2.05–2.59)	333 (0.23)	662 (0.45)	1.99 (1.75–2.27)	0.86 (0.78–0.96)
**Volvulus**	74 (0.05)	180 (0.13)	2.43 (1.86–3.19)	81 (0.05)	267 (0.18)	3.30 (2.57–4.23)	1.36 (1.07–1.72)

Legend for Table 3:

* denotes OR of constipation and concurrent condition adjusted for detection bias by dividing by the OR of concurrent condition and GERD (control group) using the method described by Friedrich. [10]

The most prevalent concurrent conditions associated with constipation (during “post–index” time period) were hemorrhoids (7.6%), diverticular disease (5.9%), ano–rectal hemorrhage (4.7%), irritable bowel syndrome (3.5%) and fecal impaction (2%). The remaining concurrent conditions had less than 1% prevalence. Colon cancer was present in 0.9% of the patients. When adjusted for detection bias as described in the methods section, the strongest association (4.4–fold) with constipation was seen for Hirschsprung's disease, followed by fecal impaction (OR 3.2). Other notable associations include anal conditions such as fissure (OR 2.5) and fistula (OR 1.7), as well as ano–rectal hemorrhage (OR 1.4) and ulcers (OR 2.1). Other concurrent conditions found to be significantly associated with constipation included hemorrhoids (OR 1.2), irritable bowel syndrome (OR 1.1), rectal prolapse (OR 1.6) and volvulus (OR 1.4). The odds of colon cancer rose by 16% in patients after the onset of constipation. Ulcerative colitis (OR 0.9) was *less* likely to be associated with constipation. No statistically significant association with constipation was seen for Crohn's disease, diverticular disease and fecal incontinence.

## Discussion

Our results suggest that chronic constipation is associated with several concurrent conditions of variable risk and prevalence and serve to eliminate the paucity of current literature on this topic, as highlighted by Talley *et al*. [11]. While a causal relationship may already exist for some of these concurrent conditions, for others, such association may provide the impetus for further research.

We note that generally the ORs were higher in the constipation cohort compared to those in the GERD cohort, although several ORs were significant in the latter as well and this may relate to increased screening and recognition prompted by the medical care received for the index condition (constipation or GERD). We found that hemorrhoids were the most prevalent concurrent condition in patients who were diagnosed with constipation and this prevalence increased by about 5% after the latter were diagnosed. Delco and Sonnenberg, in their retrospective case-control study of 96,314 veterans found that constipation was a significant co-morbidity of hemorrhoids (OR 1.48 [95% CI 1.43–1.54]) [12]; their results are very similar to ours (OR 1.24 [95% CI 1.20–1.30]). Brook *et al*., in a study of 1,215 subjects with constipation and 29,160 propensity score-matched controls, reported the prevalence of hemorrhoids to be 15.2% in the constipation group as compared to 1.5% in the control group (OR 11.8, p<0.001). [13] Our study's duration was 1 year, starting 3 months before the diagnosis of constipation and in a study design very similar to ours, Mitra *et al*., compared 48,585 subjects with 97,170 controls [14] and found the odds ratio of the association between hemorrhoids and constipation to be 4.2; this much stronger association compared to our results is likely secondary to detection bias in their study.

Ano–rectal complications such as fissures, fistulas or ulcers were rare (prevalence less than 1% each) in patients with constipation but were significantly associated with it. Brook *et al*., reported 5.8% prevalence of ‘ano–rectal conditions’ [13]; this likely includes a combined prevalence of the ano–rectal concurrent conditions that we reported separately and thus probably reflects similar prevalence. Mitra reported significant association of constipation and anal fissures (OR 5.0) and rectal ulcers (OR 4.8) without specifying their prevalence. [14] Ano–rectal hemorrhage had a relatively high prevalence in both the constipation as well as the control groups in our study, likely reflecting the similarly high prevalence of hemorrhoids and/or diverticular disease in general. Even then, the risk of ano–rectal hemorrhage was 36% higher in patients with constipation.

Diverticular disease has been proposed to be secondary to small stool volume, longer transit time as well as abnormal colonic motility and thus has been felt to be associated with constipation. [15], [16] Indeed, in our previous study [17], we reported -in the same group of patients as the current study- an odds ratio of 2.8 for this association. However, in the current study, when we accounted for possible detection bias, this association was rendered non–significant (OR 1.04 [95% CI 1.00–1.08]). Chronic constipation may be related to rectal neurological dysfunction [18] and lead to fecal impaction. Mitra demonstrated a 6.6–fold increased odds of fecal impaction in constipated patients, [14] which is similar to what we found (OR 5.6); however, the adjusted odds ratio was less, 3.2, and still significantly high. Hirschsprung's disease is a known cause of chronic constipation in adults; [19], [20] such cases are believed to be either less severe or zonal forms of colonic aganglionosis. In our study, even though it was very rare in patients with constipation (0.04%), it was the concurrent condition with the strongest association (OR 4.4 [95% CI 2.5–7.9]).

A small study involving 55 elderly patients with fecal impaction revealed impaired ano–rectal sensation in the subjects as compared to controls, preventing conscious contraction of the external anal sphincter when the internal sphincter was relaxed, thereby causing fecal incontinence. [21] This was subsequently confirmed in a much larger study of 16,331 nursing home residents with fecal incontinence and it was shown that chronic constipation increased the odds of fecal incontinence by 30–40% (2–year cross-sectional survey). [22] However, both of these studies were done in selected populations, with resulting strong possibility of selection bias. A population–based study using the Rome ll criteria for diagnosis and involving an age–stratified random sample of 507 women in Olmstead county concluded that constipation did not increase the odds of fecal incontinence (OR 1.1 [95% CI 0.8–1.5]). [23] The result from our population–based study concurs with that of this last study (OR 1.16 [95% CI 0.99–1.35]). It also follows from our results that, since constipation is associated with increased odds of fecal impaction but not fecal incontinence, constipation is not a confounder of the association between fecal impaction and incontinence. We found increased odds (63% higher) of rectal prolapse in patients with chronic constipation, consistent with prior studies [24] and likely resulting from long–term straining.. [25] Volvulus, similarly, was significantly associated with constipation in our study (36% higher odds), although not to the extent reported by Mitra (OR 10.3). [14]

Constipation has been linked with colon cancer in previous studies. A case–control study of 424 incident cases from Seattle found that constipation present for 10 years before the index date (2 years before diagnosis of colon cancer) resulted in an adjusted relative risk of 2.0 (95% CI 1.2–3.6) for colon cancer; the risk associated with the use of commercial laxatives was nullified after adjustment for constipation. [26] In a population–based case–control study of 643 cases from North Carolina, the adjusted odds ratio of colon cancer and constipation was 2.36 (95% CI 1.41–3.93). [27] The Miyagi cohort study of 41,670 individuals from Japan noted the multivariate relative risk of colon cancer in those with constipation to be 1.35 (95% CI 0.99–1.84). [28] These studies support our finding that the odds of colon cancer in constipated patients were higher than those without (OR 1.16 [95% CI 1.05–1.30]).

There are several strengths of our study. It involves a very large sample size, is population–based and, importantly, controls for detection bias (described in Methods section). In addition, usage of the Medi–Cal population for conducting this study has several advantages of its own: the drop-out rate (loss of eligibility) is significantly less than private payer plans; patients do not drop-out when they qualify for Medicare (since Medi–Cal pays for Medicare deductibles); there is a high representation of minority populations; and records from Medicare are obtained on all patients who have dual-eligibility and merged within the Medi–Cal datasets. Several limitations also apply to our study and its results. Given a different study population and design, our results may not be comparable to those reported in previous studies. We cannot establish causality based only on the strength of the associations that we observed. As is true for research conducted using an administrative database, the identification of cases and controls as well as the associated concurrent conditions are dependent on the accuracy of the claims submitted for them. However, this may not be a significant problem in our study as an audit of Medi-Cal claims found that 96.4% were medically necessary, billed appropriately and were in concordance with the data in the claims files. [8] Furthermore, as our study sample was derived from a Medicaid population, it represents data from people who are typically sicker and less affluent, thus potentially limiting the generalizability of our findings to the Medicaid population only. It is possible that diet, lifestyle-changes or treatment for CC might affect the association of the comorbidities with CC. Finally, we cannot rule out the possibility of detection bias persisting in our study; however, this is unlikely to affect the adjusted odds–ratio, thus our results are likely to remain valid.

In summary, we have reported the prevalence and strength of association of various concurrent conditions of constipation. Our findings would hopefully help direct future research in patients with chronic constipation and eventually improve patient care.
